# NCAPG Is a Promising Therapeutic Target Across Different Tumor Types

**DOI:** 10.3389/fphar.2020.00387

**Published:** 2020-04-02

**Authors:** Cuicui Xiao, Jiao Gong, Yusheng Jie, Jing Cao, Zhongcheng Chen, Rong Li, Yutian Chong, Bo Hu, Qi Zhang

**Affiliations:** ^1^ Cell-Gene Therapy Translational Medicine Research Center, Key Laboratory of Liver Disease of Guangdong Province, Guangzhou, China; ^2^ Department of Laboratory Medicine, Third Affiliated Hospital, Sun Yat-sen University, Guangzhou, China; ^3^ Department of Infectious Diseases, Key Laboratory of Liver Disease of Guangdong Province, Third Affiliated Hospital of Sun Yat-sen University, Guangzhou, China

**Keywords:** RNA sequencing, pan-cancer analysis, The Cancer Genome Atlas; Non-SMC condensin I complex subunit G, hepatocellular carcinoma weighted gene co-expression network analysis, bioinformatic analysis

## Abstract

**Background:**

With the advent of CRISPR-Cas9 genome editing tool in gene therapy, identification of aberrantly expressed genes is of great value across various cancer types. Since a large number of patients may benefit from molecular targeted gene therapy. The purpose of this study was to identify aberrantly expressed genes across various cancer types, analyze prospective mechanisms and their correlation with survival outcomes.

**Results:**

NCAPG was highly expressed in The Cancer Genome Atlas (TCGA) database, which includes the transcriptomes of 6,647 cancer and 647 normal tissue samples from 16 cancer types. Furthermore, a predicted NCAPG overexpression rate was also observed at the protein level in 16 tumor types. Importantly, high NCAPG level was significantly associated with unfavorable survival in various cancer types such as hepatocellular carcinoma (HCC), breast, lung or ovarian cancer. The multivariate analyses demonstrated that NCAPG, TNM, and Barcelona Clinic Liver Cancer (BCLC) staging were independent risk factors for mortality of patients with HCC. Moreover, functional and pathway enrichment analysis suggested that NCAPG was closely correlated with the pathways of cell cycle, cellular senescence, and mismatch repair. By weighted gene co-expression network analysis (WGCNA), we identified NCAPG as a hub gene in the turquoise module mostly related to the survival time of HCC samples.

**Conclusion:**

To our knowledge, this study represents a comprehensive RNA-Seq analysis of several tumor types, revealing NCAPG as a promising molecular target. NCAPG overexpression may play important roles in carcinogenesis and progression of tumors *via* regulating tumor-related pathways, thereby broadening the understanding of the pathogenic mechanisms and highlighting the possibility of developing novel targeted therapeutics.

## Introduction

In personalized medicine, identification of tumor-specific target or tumor-related features to improve the therapeutic potential of tumor patients is of great importance (Andre et al., 2014). Although many molecularly targeted agents that attenuate specific oncogenic driver pathways have been developed to facilitate tumor treatment, not all patients can benefit because targeting driver pathways is not applicable for all tumor types. However, the CRISPR/Cas9 is a promising tool for molecular targeted gene therapy, which can activate or repress the expression of genes linked to multiple types of cancer (Sachdeva et al., 2015; Zhan et al., 2018). Therefore, the identification of aberrant gene expression across tumors is of great significance.

Pan cancer studies may be helpful in identifying differentially expressed genes that play a vital role in many cancer types (Cao and Zhang, 2016; Cava et al., 2018). A large sample size and a broad spectrum of cancer types will be favorable for the discovery of the aberrant expression genes across cancer types. The Cancer Genome Atlas (TCGA) has performed comprehensive pan-cancer molecular study of deregulated gene expression.

Non-SMC condensin I complex subunit G (NCAPG), a subunit of the condensin complex, is responsible for the condensation and stabilization of chromosomes during meiosis and mitosis (Murphy and Sarge, 2008). To date, progress on the role of NCAPG in tumors is still limited. NCAPG is abundantly expressed in HCC, castration-resistant prostate cancer and melanoma and it was showed that NCAPG promotes HCC proliferation and migration (Ryu et al., 2007; Arai et al., 2018; Liu et al., 2018; Zhou et al., 2018). Interestingly, NCAPG has been identified as a new therapeutic target for HCC by a genome-wide CRISPR cell growth screening (Wang et al., 2017).

In this study, we analyzed a large number of RNA sequencing data containing a broad spectrum of cancer types to identify aberrantly expressed genes across tumor types. NCAPG was found to be overexpressed in multiple tumor types. Furthermore, we predicted NCAPG protein overexpression for each tumor type. To date, there are few literatures on the expression and role of NCAPG in multiple tumors. Therefore, we predicted NCAPG related pathway activity using bioinformatics tools and identified NCAPG as a hub gene in the turquoise module in HCC tissue samples by WGCNA.

## Methods

### Acquisition of Expression Data

All data including gene expression profiling and clinicopathological data were collected from TCGA cohort using Linked Omics (Vasaikar et al., 2018) (http://www.linkedomics.org/login.php) and Gene Expression Profiling Interactive Analysis (GEPIA) (Tang et al., 2017) (http://gepia.cancer-pku.cn/index.html). Expression data of GSE14520 in Gene Expression Omnibus (GEO, http://www.ncbi.nlm.nih.gov/geo/) database were obtained as well. The NCAPG level was analyzed by GEPIA based upon the TCGA database, as described previously (Jie et al., 2019). The NCAPG protein level for each individual cancer and normal tissue was examined through the Human Protein Atlas (Uhlen et al., 2010; Uhlen et al., 2015). Through the Genotype-Tissue Expression (GTEx), we obtained 2,998 normal tissue samples corresponding to 12 tumor tissues (GTEx Consortium, 2015).

### Predicting Protein Overexpression of NCAPG With RNA Profiling

The percentage of samples per tumor type, including relevant subgroups, was predicted with an increased FPKM value for NCAPG, which was used as a proxy for protein overexpression. We defined the threshold as the 97.5th percentile for the FPKM values of NCAPG in the set of FPKM values of healthy tissues. For each tumor sample, NCAPG was labeled as overexpressed when the FPKM value was above the 97.5th percentile threshold as defined in the healthy tissue samples.

### The Relevant Genes of NCAPG and Functional Enrichment Analysis

The genes most relevant to NCAPG (r > 0.4) across five tumor types were calculated using Linked Omics, which is a web portal for TCGA data. The online database GEPIA is a web server for analyzing the RNA sequencing expression based on TCGA database.

The functional annotation of NCAPG related genes included the Gene Ontology (GO) analysis and Kyoto Encyclopedia of Genes and Genomes (KEGG) pathway analysis. GO terms and pathways with a *p* value < 0.05 were significant. Both GO and KEGG pathway analyses were performed by R package “cluster Profiler”. Enrichment maps visualizing the results were drawn by R software (http:///www.r-project.org/) and Bioconductor (http://bioconductor.org/).

A gene function prediction website (Gene MANIA: http://genemania.org/) was applied to construct the gene-gene interaction networks.

### Construction of the WGCNA Co-Expression Network

The WGCNA is used to construct the gene co-expression network and identify the co-expression modules using the WGCNA package in R language (Langfelder and Horvath, 2008). Co-expression methodology is typically applied to explore correlation between gene expression levels. Genes enriched in the same pathway tend to appear with a similar expression pattern (Saris et al., 20090). Therefore, the construction of a gene co-expression network is useful in identifying genes with similar biological functions. We deleted low-expressed genes in RNA-seq data of HCC and selected the most varying genes by using the variance threshold of S.D. > 0.75, resulting in 7,373 genes for network construction. The threshold of co-expression module was set as *p* < 0.05.

### Identification of Hub Genes

Hub genes, which are highly interconnected with other genes in a module, have been shown to play a critical role in tumor development. The top ranked genes in every module are thought to be hub genes. To identify the high degree genes in the protein-protein network (PPI), the Cytoscape plugin cyto Hubba was applied to do the network analysis, and the high degree genes were identified.

### Cell Culture and siRNA Transfection

Breast cancer cell MDA-MB-231 was cultured in DMED (Gibco, Thermo Fisher Scientific) containing 10% fetal bovine serum (FBS, Gibco). The siRNA of NCAPG was purchased from RIBOBIO (Guangzhou, P.R. China). The siRNA duplexes oligonucleotides were used: siNCAPG1, 5'-GGAGUUCAUUCAUUACCUU-3'; siNCAPG2, GCUGAAACAUUGCAGAAAU. The duplex of negative control (NC) siRNA does not have any target sites in human genome. Lipofectamine RNAiMAX Reagent (Life Technologies, Thermo Fisher Scientific) was used to transfect the siRNAs according to the manufacturer's instructions.

### Immunohistochemistry (IHC)

IHC array containing breast cancer and adjacent tissues was purchased from Servicebio (Wuhan, China). The standard streptavidin–biotin–peroxidase complex method was used to conduct immunostaining investigation. The concentration of NCAPG antibody (ab56382, Abcam) was 1:250. The second antibody was Dako REAL™ EnVision™ Rabbit/Mouse antibody (Dako, K5007, Glostrup, DK).

Two independent experienced pathologists analyzed the staining of this IHC array. According to the strength and area of staining, it can be divided into different grades: corresponding to no staining, weak staining, moderately positive, strong positive, the score ranged from 0-3; similarly, the area of staining also ranged from 0–3, 0 as no area staining, one as 1%–30%, two as 30%–60%, three as over 60%. IHC score is the sum of the strength and area of staining and the highest score is 9.

### Extraction of Total RNA and Quantitative Real-Time Polymerase Chain Reaction (qPCR)

Total RNA of breast cancer and MDA-MA-231 were isolated by Trizol reagents (Invitrogen, Thermo Fisher Scientific). Transcriptor First Strand cDNA Synthesis Kit (Roche Diagnostics) and SYBR Green (Roche Diagnostics) were applied for cDNA synthesis and qPCR, respectively. The sequences of primers used in this study were provided as followed: forward of NCAPG is 5'-GAAGAGGAAGATGGTGGCCT-3' and reverse is 5'-TGTTTATGAGCAGGCACACT-3'; forward of β-actin is 5'-GCACCCAGCACAATGAAGAT-3' and reverse is 5'- ACATCTGCTGGAAGGTGGAC-3'. β-actin was used as an endogenous control.

### Statistical Analysis

Graphpad prism 8 was used for statistical analysis. Data were evaluated by t-test and presented as mean ± standard deviation (x ± s). *P* < 0.05 was considered to be statistically significant.

## Results

### Predicted Protein Overexpression of NCAPG by RNA-Seq

**Figure 1:**
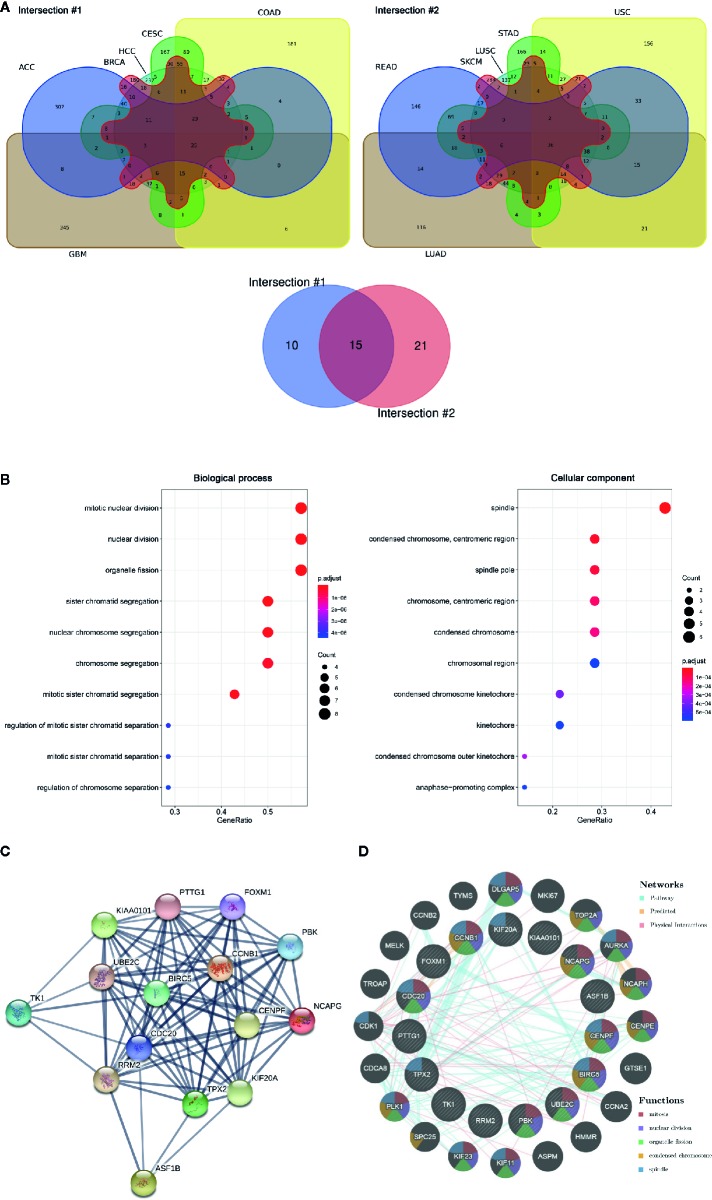
The aberrantly expressed genes identified across cancer types. **(A)** Venn diagram of deferential gene expression across various tumor types based on TCGA and GTEx projects *via* GEPIA (http://gepia.cancer-pku.cn/index.html). ACC, adrenocortical Carcinoma; BRCA, breast invasive carcinoma; CESC, cervical squamous cell carcinoma and endocervical adenocarcinoma; COAD, colon adenocarcinoma; GBM, glioblastoma multiforme; HCC, hepatoma carcinoma; LUAD, lung adenocarcinoma; LUSC, lung squamous cell carcinoma; READ, rectum adenocarcinoma; SKCM, skin cutaneous melanoma; STAD, stomach adenocarcinoma; UCS, uterine carcinosarcoma. **(B)** GO of 15 aberrantly expressed genes across tumor. Biological process, Cellular component. **(C)** PPI network was drawn using STRING online tool and the interaction score was set to high confidence (0.700). Network nodes represent proteins and edges represent protein-protein associations. **(D)** Pathway/predication/physical interaction analysis of genes according to human expression data in Gene MANIA (http://genemania.org/).

**Figure 2:**
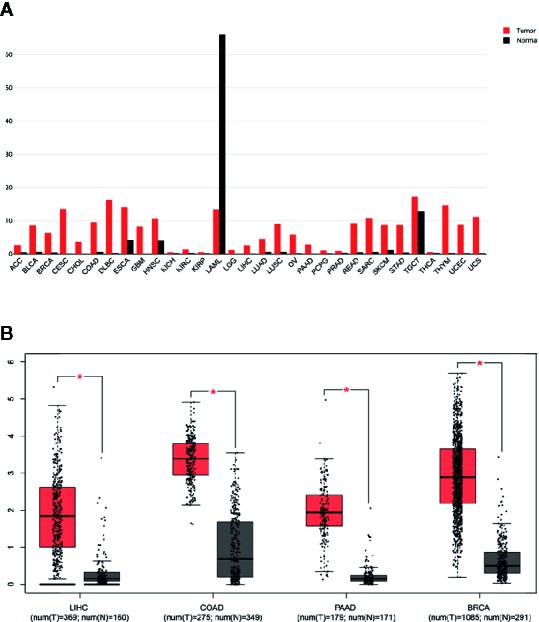
Overexpression of NCAPG in different cancer types. **(A, B)** Comparison of NCAPG expression between cancer tissues and non-cancerous tissues involved in TCGA database based on GEPIA. **(A)** The expression level of NCAPG was higher in many cancer tissues than non-cancerous tissues. Red, cancer tissues; Black, non-cancerous tissues. **(B)** Box blot showed that NCAPG expression was upregulated in cancer tissues compared with non-cancerous tissues. LIHC, Liver hepatocellular carcinoma; COAD, colon adenocarcinoma; PAAD, pancreatic adenocarcinoma; BRCA, breast invasive carcinoma; N, non-cancerous tissues; T, cancer # *p < 0.05.

The RNA sequencing expression data of 4,262 tumors and 2,998 normal samples representing 12 tumor types from the TCGA and GTEx projects were analyzed *via* GEPIA. A total of 15 genes were identified to be significantly deregulated among 12 tumor types while the top 500 most differential genes of every tumor type were selected, including NCAPG, ASF1B, BIRC5, CCNB1, CDC20, CENPF, FOXM1, KIAA0101, KIF20A, PBK, PTTG1, RRM2, TK1, TPX2, UBE2C (**Figure 1A**, **Supplementary Figure 1**). Moreover, these 15 genes were subjected to cluster Profiler for GO analysis. GO term annotation showed that these genes correlated with mitotic nuclear division, nuclear division and organelle fission (BP), spindle, spindle pole, and condensed chromosome centromeric region (CC) (**Figure 1B**). Then, protein co-expression network analysis was carried out and 13 key genes from the most network binding nodes were found, including NCAPG, BIRC5, CCNB1, CDC20, CENPF, FOXM1, KIAA0101, KIF20A, PBK, PTTG1, RRM2, TPX2, and UBE2C (**Figure 1C**). In addition, Genemania analysis also revealed these 15 genes were enriched in mitosis, nuclear division, organelle fission, condensed chromosome, and spindle signaling pathway (**Figure 1D**), thus indicating involvement in cancer progression. Therefore, we focused on NCAPG which highly correlated with condensed chromosome and mitosis. NCAPG was upregulated in multiple solid malignancies but it was downregulated in hematologic malignancies such as lymphoblastic acute myeloid leukemia (LAML) (**Figures 2A, B**). The median number of tumor samples analyzed per tumor type was 337.5 (interquartile range, 145.25 to 466.5), ranging from 57 in UCS to 1,085 in breast cancer (BRCA).

**Figure 3:**
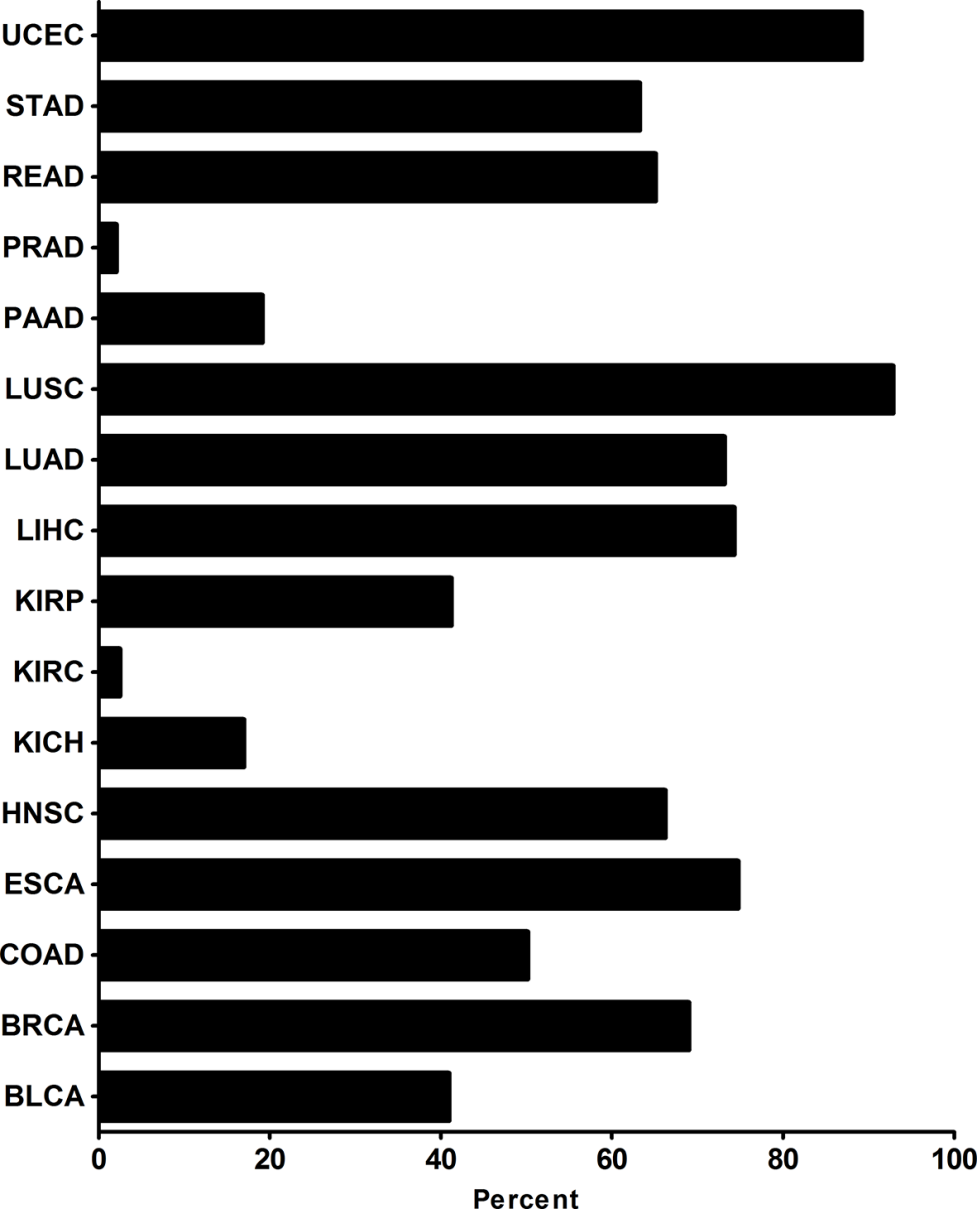
NCAPG overexpression rates in many tumor types NCAPG overexpression rates in tumor types were determined with functional RNA sequencing data according to TCGA database. The x-axis shows the percentage of samples with overexpression of NCAPG. The percentage of samples per tumor type was predicted with an increased FPKM value for NCAPG, which was used as a proxy for protein overexpression. The threshold as the 97.5th percentile for the FPKM values of NCAPG was defined in the set of FPKM values of healthy tissues. For each tumor sample, NCAPG was labeled as overexpressed when the FPKM value was above the 97.5th percentile threshold as defined in the healthy tissue samples.

By analyzing 7,294 RNA-sequencing data of 16 tumor types from the Cancer Genome Atlas (TCGA), we observed that a predicted NCAPG overexpression rate was in 74.26% of samples for HCC, 68.96% for breast cancer (BRCA), 73.10% for lung adenocarcinoma (LUAD), 92.81% for lung squamous cell carcinoma (LUSC), 50.11% for colon adenocarcinoma (COAD), 40.93% for bladder urothelial carcinoma (BLCA), 66.2% for head, neck squamous cell carcinoma (HNSC), 65.06% for rectum adenocarcinoma (READ), and 41.18% for kidney renal papillary cell carcinoma(TCGA-KIRP) (**Figure 3** and **Supplementary Table 1**). The other tumor types were excluded owing to the small sample size of normal tissues.

### High NCAPG Was a Prognostic Marker in Several Types of Tumor

**Figure 4:**
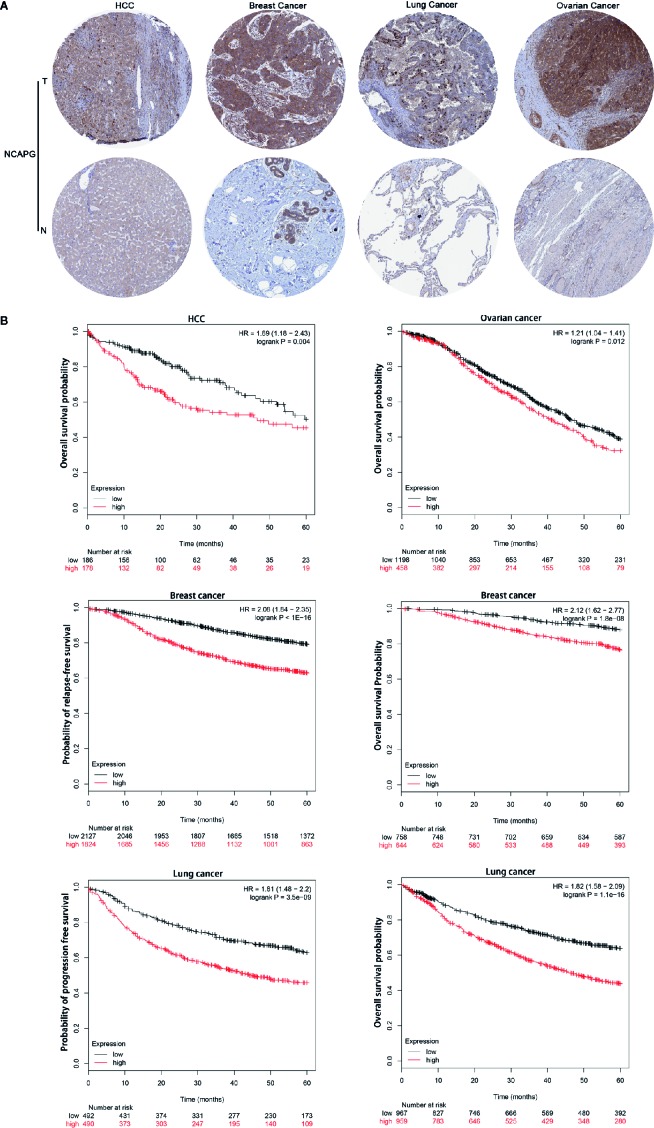
NCAPG correlated with poor survival in patients with tumors. **(A)** Immunohistochemistry data from The Human Protein Atlas (http://www.proteinatlas.org/). The results of immunohistochemistry from left to right were as follows HCC compared with normal liver tissue, breast cancer compared with normal breast tissue, lung cancer compared with normal lung tissue and ovarian cancer compared with normal ovarian tissue. NCAPG-positive cells displayed brown staining in the periphery and cytoplasm. **(B)** Determination of prognostic value of NCAPG mRNA expression *via* the Kaplan-Meier (KM) plotter. Survival curves are plotted for patients with HCC, breast cancer, lung cancer, and ovarian cancer. Red color represents tumor patients with high NCAPG expression and black color represents tumor patients with low NCAPG expression.

Representative staining patterns of NCAPG expression in HCC, breast cancer, lung cancer, and ovarian cancer by IHC were shown from Human Protein Atlas (**Figure 4A**). NCAPG cytoplasmic/nucleus staining patterns of tumor cells were displayed in various intensities. The “Kaplan-Meier plotter” (KM plotter) based upon Gene Expression Omnibus database or RNA-seq datasets was used to analyze the prognostic value of NCAPG. Kaplan Meier overall survival curves were drafted for all HCC patients (n=364, HR= 1.69, *p*=0.004), for breast cancer patients (n =1,402, HR=2.12, *p*=1.8e-08), lung cancer patients (n=1,926, HR=1.82, *p <* 0.001), and ovarian cancer patients (n =1,656, HR=1.21, *p*=0.012) (**Figure 4B**). Moreover, relapse-free survival curve for breast cancer patients (n=3,951, HR=2.08, *p*< 0.001) and progression free survival curve for lung cancer patients (n=982, HR=1.81, *p*< 0.001) were also obtained. These data suggest that high expression of NCAPG correlated with shorter survival in patients with several types of cancer, including HCC, breast cancer, lung cancer, and ovarian cancer.

**Figure 5:**
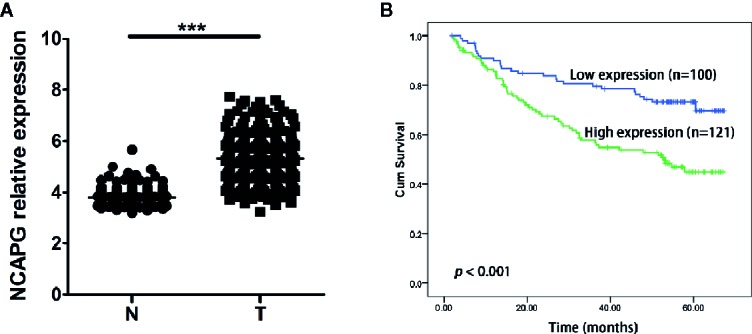
High NCAPG level correlated with poor survival in HCC. **(A)** Comparison of NCAPG expression between HCC cancer tissues and non-cancerous tissues with GEO dataset. The gene of NCAPG is significantly upregulated in HCC cancer tissues. N, normal; T, tumor. ***p < 0.0001. **(B)** Prognostic value of NCAPG mRNA expression with GEO dataset. Survival curves are plotted for patients with HCC. Compared with low NCAPG expression, the overall survival rates of patients with high NCAPG expression was lower. Blue color represents HCC patients with high NCAPG expression and green color represents HCC patients with low NCAPG expression. *p* < 0.001.

Furthermore, we validated the expression pattern of NCAPG with microarray data retrieved from GEO (GSE14520, n=471, **Figure 5A**). To analyze the association of NCAPG with clinical and pathological features on 221 clinical cases, X-tile software was used to determine the optimal cut-off values as previously described (Jie et al., 2019). Patients were divided into low group (NCAPG ≤ 5.1) and high group (NCAPG >5.1) for further analysis (GSE14520, n=221). The expression of NCAPG seemed to differ substantially between some indicated features, including cirrhosis, TNM staging, AFP level (**Supplementary Table 2**). The overall survival rate was significantly lower in patients with high NCAPG expression than in those with low NCAPG expression (*p* < 0.001; **Figure 5B**). Next, Cox proportional hazards regression analysis was performed to validate whether the NCAPG was confounded by underlying clinical conditions. A univariate analysis revealed that the NCAPG was a significant predictor of overall survival (*p* = 0.01, **Table 1**). The multivariate analyses demonstrated that NCAPG, TNM, and BCLC were independent risk factors for mortality of patients with HCC (*p* = 0.011, **Table 1**).

**Table 1:** Univariate and multivariate analysis of factors associated with overall survival ^a^.

Clinical Variable	Hazard Ratio (95% CI ^c^)	P Value
**Univariate analysis** ^**b**^		
NCAPG (High vs Low)	1.8 (1.1–2.7)	**0.010**
Gender (M vs F)	1.7 (0.8–3.5)	0.154
Age (> 45 vs ≤45)	1.1 (0.7–1.7)	0.770
ALT (≥50 vs <50 U/L)	1.1 (0.7–1.7)	0.726
TNM (II/III vs I)	3.0 (1.8–4.9)	**<0.001**
BCLC (B + C vs 0+A)	3.5 (2.3–5.5)	**<0.001**
AFP (≥300 vs <300 ng/ml)	1.6 (1.1–2.4)	**0.042**
Cirrhosis (Yes vs No)	4.6 (1.1–18.8)	**0.032**
**Multivariate analysis** ^**d**^		
NCAPG (High vs Low)	1.8 (1.2–3.0)	**0.011**
TNM (II/III vs I)	1.9 (1.1–3.3)	**0.029**
BCLC (B + C vs 0+A)	2.5 (1.5–4.1)	**<0.001**

Bold indicates significant values.

a Analysis was performed on the entire cohort (n = 221).

b Univariate analysis, Cox proportional hazards regression.

c 95% CI, 95% confidence interval.

d Multivariate analysis, Cox proportional hazards regression.

### Relevant Genes of NCAPG and Functional Annotation of NCAPG

**Figure 6:**
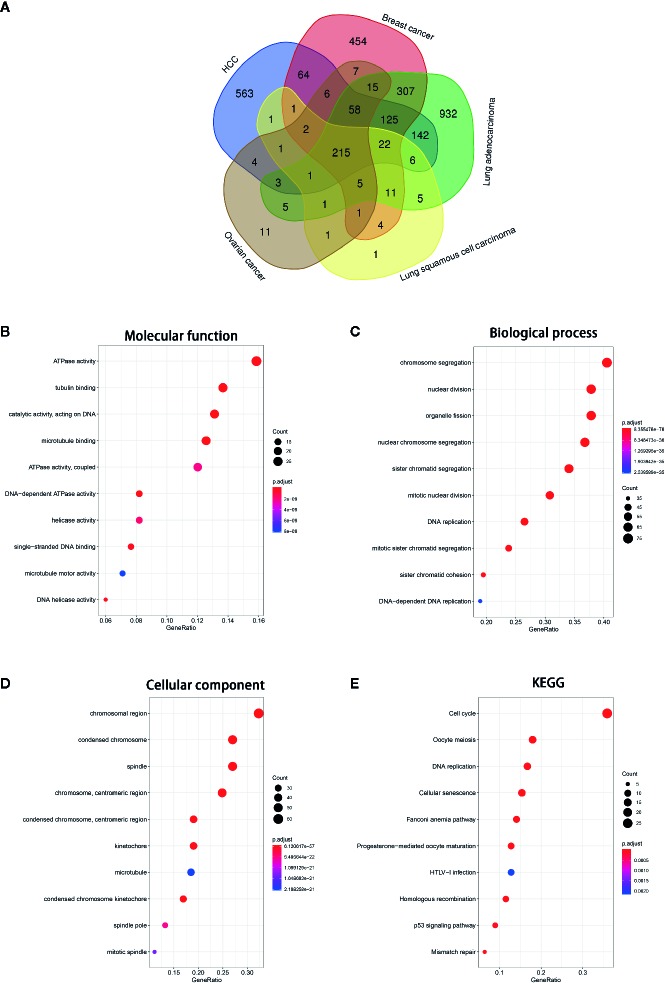
GO and KEGG pathway of the genes relevant to NCAPG. **(A)** Venn diagrams of the genes relevant to NCAPG in various tumor types based on TCGA database *via* Linked Omics. The genes most relevant to NCAPG (r > 0.4) across five tumor types were calculated. **(B–D)** Molecular function, Biological process, Cellular component. The size of the colored dots represents the enriched number of genes in each GO classification. The red dots indicate high enrichment and blue dots indicate low enrichment. The FDR value is expressed by the color order on the right edge. **(E)** KEGG pathway enrichment of NCAPG. The names of top 10 KEGG pathway were indicated by the y-axis. The size of dot in KEGG pathway bubble plot shows the enriched genes. High enriched represented by red, otherwise, by blue.

To investigate the function of NCAPG in tumors, we first identified genes most relevant to NCAPG (r > 0.4) across five types of tumor using Linked Omics. The common relevant genes of NCAPG were analyzed based upon 3,091 tumor samples, including HCC (n=377), breast cancer (n=1,097), lung adenocarcinoma (n=522), lung squamous cell carcinoma (n=504), and ovarian cancer (n=591) expression profiling datasets, respectively (**Figure 6A**). Then these 215 relevant genes were subjected to cluster Profiler for GO and KEGG pathway analysis. GO categories enrichment analysis showed that these genes were mainly enriched in ATPase activity, tubulin binding and catalytic activity, acting on DNA (MF), chromosome segregation, nuclear division and organelle fission (BP), chromosomal region, condensed chromosome and spindle (CC) (**Figures 6B–D**). Furthermore, the KEGG pathway annotation revealed that cell cycle, oocyte meiosis, DNA replication, cellular senescence and mismatch repair were the most significantly enriched pathways (**Figure 6E**).

### Construction of Weighted Gene Co-Expression Modules

**Figure 7:**
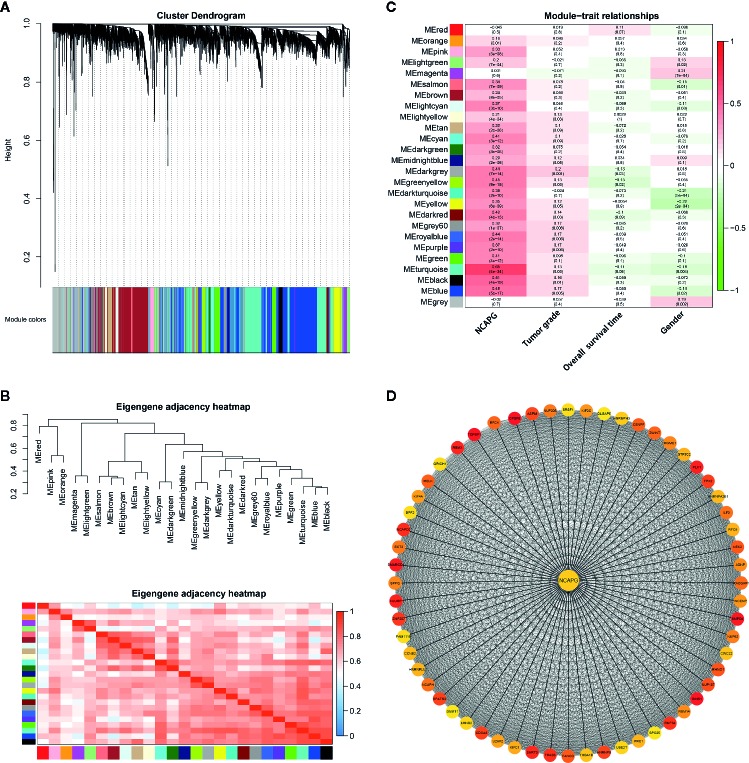
Gene modules identified by WGCNA. **(A)** Network analysis of gene expression in HCC identified 25 distinct modules of co-expression genes. The dendrogram produced by average linkage hierarchical clustering of 7,373 genes based on WGCNA package in R. Each colored row indicates a color-coded module which includes a group of highly connected genes. **(B)** Heatmap plot of the adjacencies of modules. Red represents high adjacency (positive correlation) and blue represents low adjacency (negative correlation). **(C)** Heatmap of the correlation between module eigengenes and different clinical information of HCC (tumor grade, overall survival time and gender). The module name is shown on the left side of each cell. Numbers in the table report the correlations of the corresponding module eigengenes and stage, with the *p* values printed below the correlations in parentheses. **(D)** PPI network of genes in the turquoise module. PPI network was drawn using Cytoscape 3.6 software. Network nodes represent proteins and edges represent protein-protein associations.

Two hundred and sixty-eight HCC samples with clinical data were obtained in co-expression analysis *via* WGCNA (**Supplementary Figure 2**). In this study, we have chosen the soft threshold power of β = 6 to ensure a scale-free network (**Supplementary Figure 3**). A total of 25 modules was identified, and the connectivity of eigengenes was analyzed (**Figures 7A, B**). Moreover, there were multiple modules related to one or more clinical traits, such as tumor stages, overall survival time, and gender. As shown in **Figure 7C**, the light yellow, midnight blue, dark green, green yellow, yellow, dark red, grey60, royal blue, purple, turquoise, black, and blue modules were related to three tumor stages; dark green, and green yellow modules were negatively related to the overall survival time, and turquoise module slightly exceeded signiﬁcance level; magenta, salmon, dark turquoise, yellow, turquoise, blue, and grey modules were related to gender. Taking into account gender disparity in HCC, we selected the turquoise module which related to different HCC stages, shorter overall survival time, gender for further analysis. Then these 1,576 genes in turquoise module were conducted to clusterProfiler for GO and KEGG pathway analysis (**Supplementary Figure 4**). Consistent with the results mentioned above, cell cycle, oocyte meiosis, DNA replication and mismatch repair were the most significantly enriched pathways by KEGG pathway annotation.

To explore the interaction between the 1,576 genes in the turquoise module, PPI network was explored and visualized by Cytoscape (**Supplementary Figure 5**). In the PPI network, genes with a connectivity degree of ≥ 10 were also defined hub genes. The degree of connectivity to NCAPG was 727. Furthermore, the high degree genes were calculated by the cytohubba plugin. The co-expression network of top 60 ranked genes for the turquoise module was constructed as shown in **Figure 7D**. Importantly, NCAPG was identified as the hub gene in the turquoise module.

### NCAPG Was Upregulated in Breast Cancer and Affected Cell Proliferation

**Figure 8:**
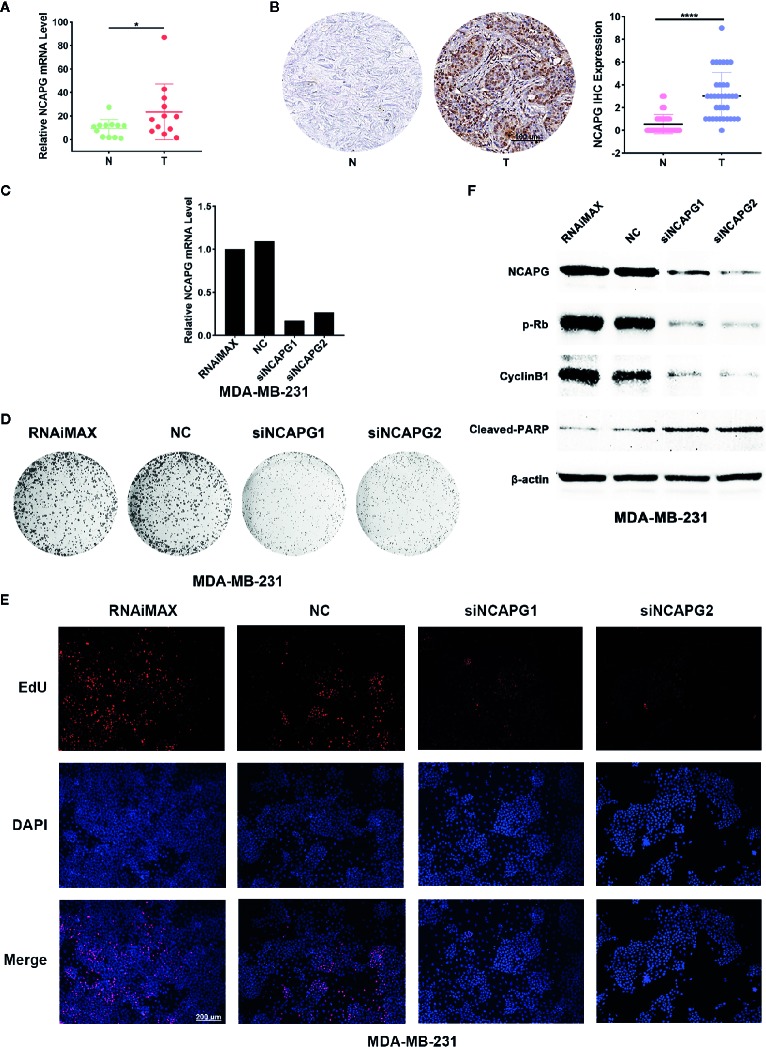
NCAPG was upregulated in breast cancer and affected the Proliferative ability of cancer cell. **(A)** High mRNA expression of NCAPG in breast cancer. n=12. *p < 0.05. N, Non-neoplastic tissue adjacent to cancer; T, cancer. **(B)** The protein level of NCAPG was up regulated in breast cancer. The representative IHC for NCAPG in breast cancer was showed (left panel) and quantifications of 35 paired breast cancer and non-tumor tissues as indicated (right panel). ****p < 0.0001. N, Non-neoplastic tissue adjacent to cancer; T, cancer. **(C)** The mRNA level of NCAPG was decreased by NCAPG siRNAs in breast cancer cell. Forty-eight hours after transfection with siNCAPGs or NC, the expression of endogenous NCAPG was analyzed by qPCR. β-Actin served as an internal control. **(D)** The representative images of colony formation assay of MDA-MB-231 cells treated with NCAPG siRNAs. **(E)** EdU staining of MDA-MB-231 cells transfected with NCAPG siRNAs. **(F)** Inhibition of NCAPG expression can reduce phosphorylated retinoblastoma protein (pRb) and cyclin B1 level but upregulates cleaved-PARP. Twenty-four hours after transfection, MDA-MB-231 cells were deprived of serum for 48 h, and then the levels of endogenous proteins were detected using western blotting. β-Actin was used as an internal control.

To verify the expression of NCAPG in tumors, we extracted mRNA from 12 pairs of breast cancer and the normal tissue adjacent to the cancer to verify the expression of NCAPG. The qPCR results showed NCAPG was upregulated in breast cancer (**Figure 8A**), which was consistent with analysis of TCGA data. Additionally, we found that the protein of NCAPG was upregulated in breast cancer (**Figure 8B** left panel). Statistical results of 35 pairs of breast cancer tissue microarray staining showed that NCAPG was upregulated in tumor tissues (**Figure 8B** right). To investigate whether high expression NCAPG plays a crucial role in breast cancer, we suppressed NCAPG expression by siRNAs in MDA-MB-231. qPCR was used to detect the inhibition efficiency of siNCAPG1 and siNCAPG2.The results showed that NCAPG was greatly decreased by the siRNAs of NCAPG (**Figure 8C**). Then we performed colony formation assay and found that inhibition of NCAPG expression the number of clones in MDA-MB-231 cells was reduced (**Figure 8D**). Moreover, EdU assay showed that inhibiting NCAPG could significantly reduce MDA-MB-231 cells proliferation (**Figure 8E**). Furthermore, knockdown of NCAPG increased cleaved-PARP protein level but decreased phosphorylated levels of retinoblastoma protein (pRb) and cyclin B1 protein (**Figure 8F**).

## Discussion

We performed a comprehensive analysis of the TCGA tumor database based on 7,294 clinical samples and identified overexpression of NCAPG across tumor types. Next, we predicted NCAPG overexpression rate in multiple tumor types and found NCAPG was consistently unregulated. Furthermore, NCAPG was a prognostic marker in several types of tumor, including HCC, breast cancer, lung cancer, and ovarian cancer. More importantly, the multivariate analyses demonstrated that NCAPG, TNM and BCLC staging were independent risk factors for mortality in patients with HCC with GEO dataset. In addition, using bioinformatics analysis, we identified that NCAPG correlated with ATPase activity, tubulin binding and catalytic activity, acting on DNA (MF), chromosome segregation, nuclear division and organelle fission (BP), chromosomal region, condensed chromosome and spindle (CC) and was involved in cancer-related signaling pathways, cell cycle, DNA replication and mismatch repair across different cancer types. Moreover, NCAPG was identified as a hub gene in HCC samples using WGCNA. Moreover, consistent with analysis of TCGA data, both the mRNA and protein levels of NCAPG were upregulated in breast cancer tissues compared with adjacent tissues. Furthermore, knockdown of NCAPG by siRNA significantly decreased cell proliferation in breast cancer cell line MDA-MB-231.

In our pan-cancer analysis, in high-incidence tumor types such as BRCA, LUAD and LUSC, more than 50% of samples had overexpressed NCAPG expression. Emphasis on upregulated NCAPG can be a promising new strategy for personalized treatment. More importantly, the CRISPR/Cas9 is a promising tool for molecular targeted gene therapy, which can activate or repress the expression of genes linked to multiple types of cancer. The work of Yu Wang *et al*. shows that NCAPG is a true target identified by CRISPR for HCC tumor cell growth (Wang et al., 2019). This approach might pave the way for molecular targeted gene therapy in the near future to treat tumors with specific features, such as NCAPG overexpression, irrespective of their origin, and location. Because elevated NCAPG level was a regular event in the large set of tumors we analyzed, targeted treatment options might be available for NCAPG. This means that a large number of patients could possibly benefit from targeted inhibition of NCAPG. We believe that the comprehensive pan-cancer molecular study of a very large set of samples representing many tumor types is worthy of further investigation and may provide better treatment options for a large number of patients.

Cell cycle and DNA damage response pathway are frequently mutated in cancer (Kastan and Bartek, 2004; Strzyz, 2016). For the first time, the US Food and Drug Administration approved a cancer drug for treatment based on a tumor biomarker and not the tumor's original location: pembrolizumab, indicated for treatment of mismatch repair deficient, or microsatellite instability-high advanced solid tumors (Boyiadzis et al., 2018). It has been reported that the knockdown of NCAPG expression could not only reduce HCC cell viability, but also induce apoptosis and arrest the cells at the S phase of the cell cycle by regulating the expression of Bax, cleaved caspase-3, E-cadherin, cyclin A1, CDK2, Bcl-2, N-cadherin, and HOXB9 (Wang et al., 2019). Downregulation of NCAPG by miR-99a-3p inhibits cancer cell aggressiveness *via* decreasing cell proliferation, migration, and invasion in castration-resistant prostate cancer (Arai et al., 2018). Consistent with these findings, our research also showed that NCAPG might regulate cancer-related signaling pathways, cell cycle, DNA replication, and mismatch repair across cancer types. Therefore, NCAPG is a promising target for cancer therapy across cancer types.

NCAPG might promote tumor development by dysregulating the cell cycle, mismatch repair and cellular senescence. As a result, we suggest that the overexpression of NCAPG across tumor types is associated with poor prognosis by regulating the above-mentioned pathways. According to the findings above, it can be concluded that NCAPG may be involved in carcinogenesis and tumor growth across different cancer types.

There are some drawbacks concerning RNA sequencing expression data, which is used as a screening tool to evaluate differential gene expression across a very large number of samples consisting of several tumor types. Expression of a gene can be affected at many levels, including mRNA stability, translation, and post-transcriptional control such as miRNA-mediated regulation of mRNA stability (du Toit, 2016; Franks et al., 2017). However, RNA sequencing data does shed light on answering questions concerning NCAPG overexpression across tumors through a more efficient method rather than large-scale lmmunohistochemistry (IHC) analyses, which is most widely used in the clinic to determine protein expression. Hence, subsequent IHC validation might be needed.

In conclusion, analysis of larger sample sizes across tumor types will also enable the scientists to identify the deregulated genes that are important in driving cancer. This integrative analysis has identified aberrantly expressed NCAPG across the 16 tumor types and identified specific signaling pathways regulated by NCAPG. Patients with high NCAPG levels in several cancer types correlated with short survival time through regulating cancer related pathways. Moreover, NCAPG was a hub gene in the turquoise module in HCC tissue.

## Conclusions

In conclusion, this study represents a comprehensive RNA-Seq analysis of several types of tumor revealing NCAPG as a promising molecular target and NCAPG overexpression may play important roles in carcinogenesis and progression of tumors through regulating tumor-related pathways, including cell cycle, cellular senescence, and mismatch repair. Moreover, high NCAPG level significantly correlated with poor survival in patients with several types of cancer, including HCC, breast cancer, lung cancer, and ovarian cancer. The multivariate analyses demonstrated that NCAPG, TNM, and BCLC staging were independent risk factors for mortality of patients with HCC with GEO dataset.

## Data Availability Statement

All data including gene expression profiling and clinicopathological data were collected from TCGA cohort using LinkedOmics (http://www.linkedomics.org/login.php) and Gene Expression Profiling Interactive Analysis (GEPIA) (http://gepia.cancer-pku.cn/index.html). Expression data of GSE14520 in Gene Expression Omnibus (GEO, http://www.ncbi.nlm.nih.gov/geo/) database were obtained as well.

## Ethics Statement

This study was approved by the Institute Research Ethics Committee of the Third affiliated hospital of Sun Yat-sen University and was performed in accordance with the approved guidelines.

## Author Contributions

YC, BH, and QZ designed the research and analyzed the data. CX, JG, YJ, and RL analyzed data and wrote the paper. JC and ZC reviewed the clinical information. All authors approved the final version.

## Funding

This work was supported by a grant from National Natural Science Foundation of China (31401095, 81670601, 81870449, 81773176, 81802897), Natural Science Foundation of Guangdong Province (2016A030313250, 2014A030313095), Science and Technology Program of Guangzhou (201804010474), and Medical Scientific Research Foundation of Guangdong Province (A2017118) Special fund for frontier and key technology innovation of Guangdong Province (2015B020226004).

## Conflict of Interest

The authors declare that the research was conducted in the absence of any commercial or financial relationships that could be construed as a potential conflict of interest.

## Abbreviations

NCAPG, Non-SMC condensin I complex, subunit G; GEO, Gene Expression Omnibus Datasets; TCGA, The Cancer Genome Atlas database; WGCNA, weighted gene co-expression network analysis.
