# Supplementary material for: NCAPG Is a Promising Therapeutic Target Across Different Tumor Types
Source: Front Pharmacol. 2020 Apr 2;11:387. doi: 10.3389/fphar.2020.00387 (PMC7142249; doi:10.3389/fphar.2020.00387)
Supplement: Supplementary file 1 [file DataSheet_1.docx]

**Supplementary Tables**

Supplementary Table 1. TCGA cancer dataset

|  | Tumor sample (n) | Normal sample (n) |
| --- | --- | --- |
| Bladder Urothelial Carcinoma(TCGA-BLCA) | 408 | 19 |
| Breast Invasive Carcinoma(TCGA-BRCA) | 1092 | 113 |
| Colon Adenocarcinoma(TCGA-COAD) | 456 | 41 |
| Esophageal Carcinoma(TCGA-ESCA) | 162 | 11 |
| Head and Neck Squamous Cell Carcinoma(TCGA-HNSC) | 500 | 44 |
| Kidney Chromophobe(TCGA-KICH) | 65 | 24 |
| Kidney Renal Clear Cell Carcinoma(TCGA-KIRC) | 530 | 72 |
| Kidney Renal Papillary Cell Carcinoma(TCGA-KIRP) | 289 | 32 |
| Liver Hepatocellular Carcinoma(TCGA-LIHC) | 373 | 50 |
| Lung Adenocarcinoma(TCGA-LUAD) | 513 | 59 |
| Lung Squamous Cell Carcinoma(TCGA-LUSC) | 501 | 49 |
| Pancreatic Adenocarcinoma(TCGA-PAAD) | 178 | 4 |
| Prostate Adenocarcinoma(TCGA-PRAD) | 496 | 52 |
| Rectum Adenocarcinoma(TCGA-READ) | 166 | 10 |
| Stomach Adenocarcinoma(TCGA-STAD) | 375 | 32 |
| Uterine Corpus Endometrial Carcinoma(TCGA-UCEC) | 543 | 35 |

| Supplementary Table 2. Association of NCAPG expression with clinical features | | | |
| --- | --- | --- | --- |
| Variable | *N* | *P* value ^a^ |  |
| Gender |  |  |  |
| Male | 191 | 0.490 |  |
| Female | 30 |  |  |
| Age |  |  |  |
| ≤45 | 154 | 0.478 |  |
| >45 | 67 |  |  |
| Cirrhosis |  |  |  |
| Yes | 203 | **0.049** |  |
| No | 18 |  |  |
| TNM |  |  |  |
| II/III | 126 | **0.001** |  |
| I | 93 |  |  |
| No data | 2 |  |  |
| BCLC |  |  |  |
| B + C | 51 | 0.062 |  |
| 0+A | 168 |  |  |
| No data | 2 |  |  |
| AFP |  |  |  |
| <300 ng/ml | 118 | **0.027** |  |
| ≥300 | 103 |  |  |
| ALT |  |  |  |
| <50 U/L | 91 | 0.099 |  |
| ≥50 | 130 |  |  |
| ^a^: NCAPG high expression vs NCAPG low expression; Bold indicates significant values. | | | |


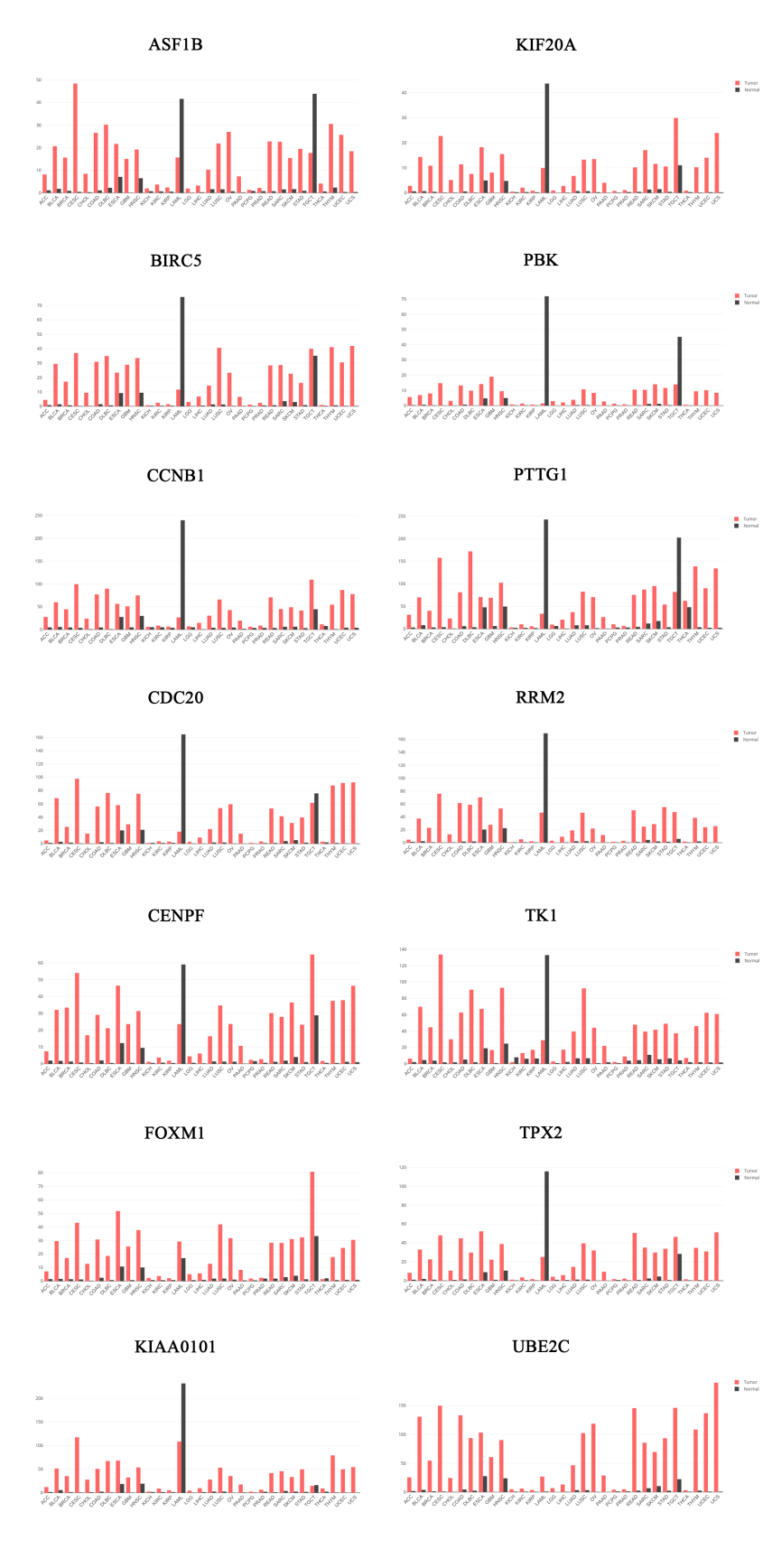


**Supplementary Fig. 1 Comparison of gene expressions between cancer tissues and non-cancerous tissues.**

Comparison of 14 gene expressions between cancer tissues and non-cancerous tissues involved in TCGA database based on GEPIA.


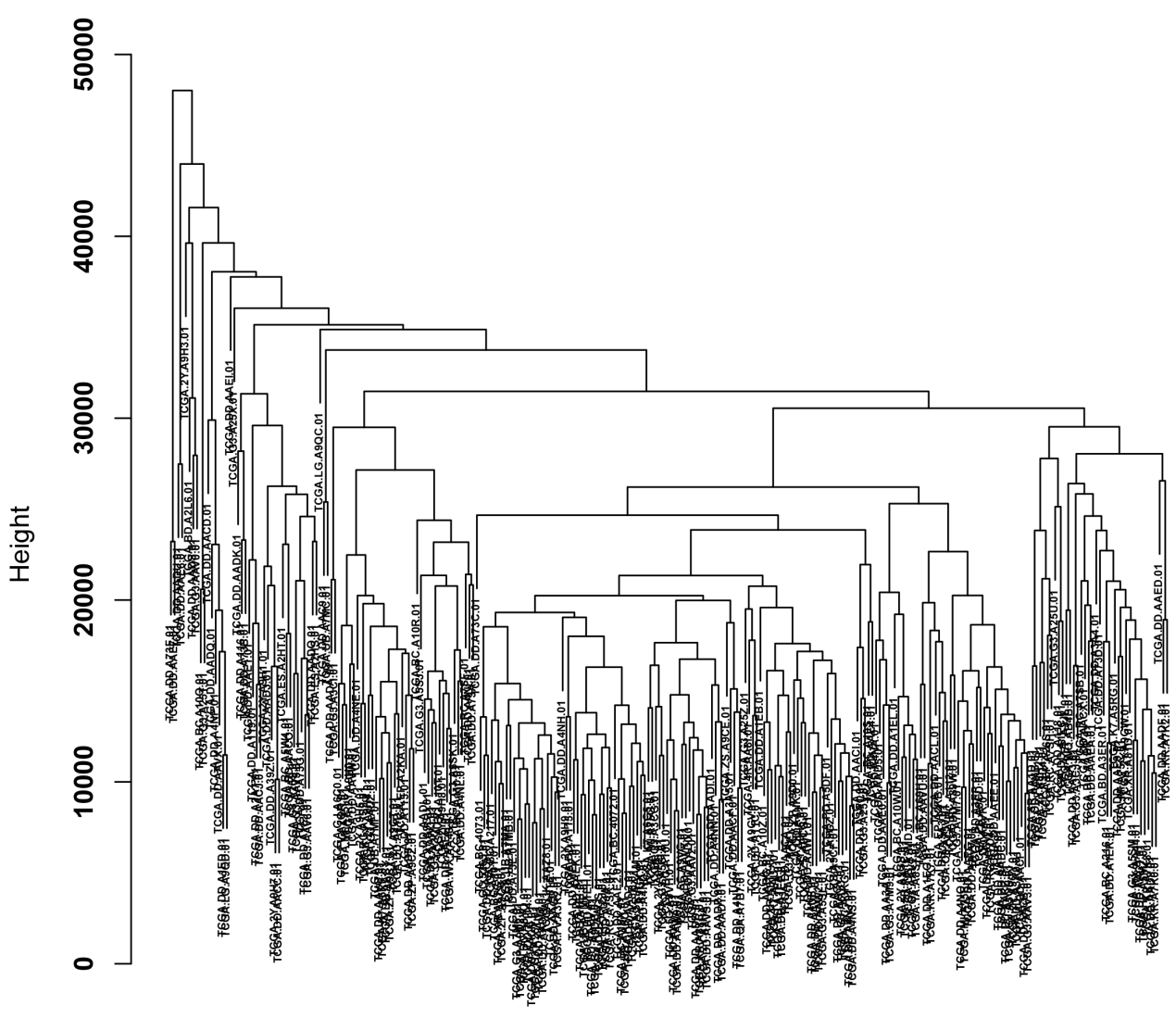


**Supplementary Fig. 2 Clustering dendrogram of 268 tumor samples.**

The clustering was based on the expression data of genes among tumor samples in HCC.


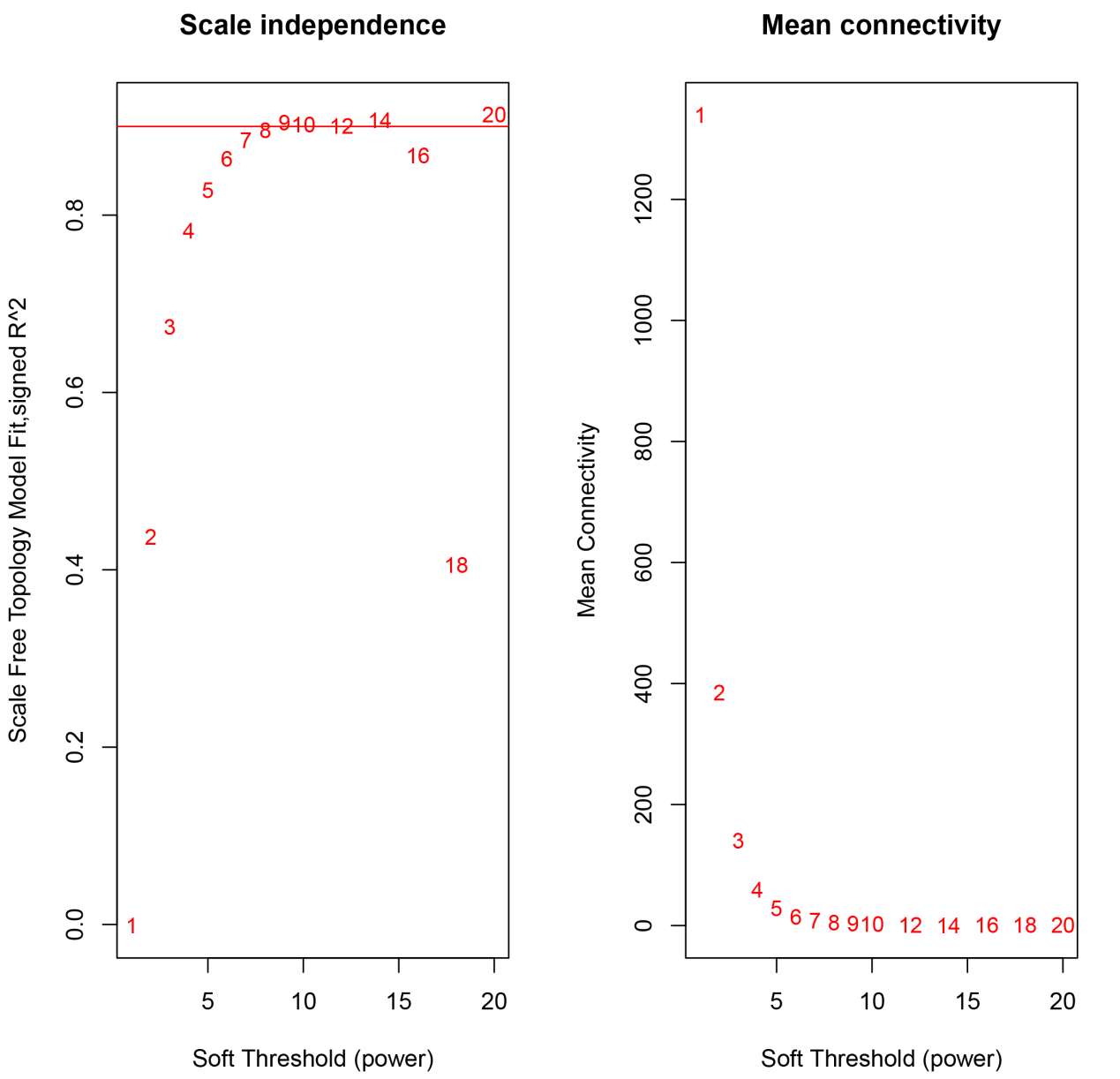


**Supplementary Fig. 3 Determination of soft-thresholding power in the weighted gene co-expression network analysis (WGCNA)**

Analysis of the scale-free fit index for various soft-thresholding powers (β).


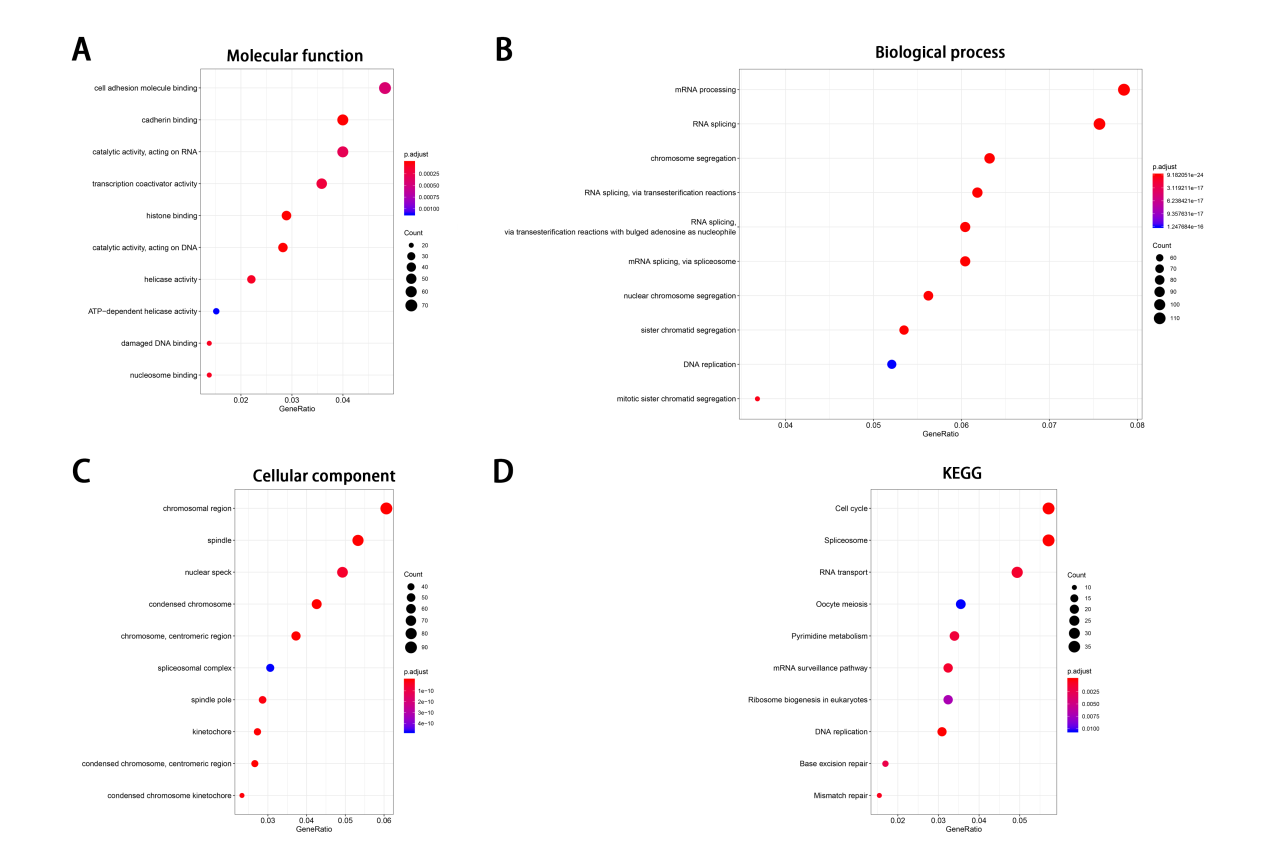


**Supplementary Fig. 4 Gene Ontology (GO) and KEGG pathway of the genes in the turquoise module.**

(A, B, C) Biological process. (D) KEGG pathway enrichment of NCAPG.


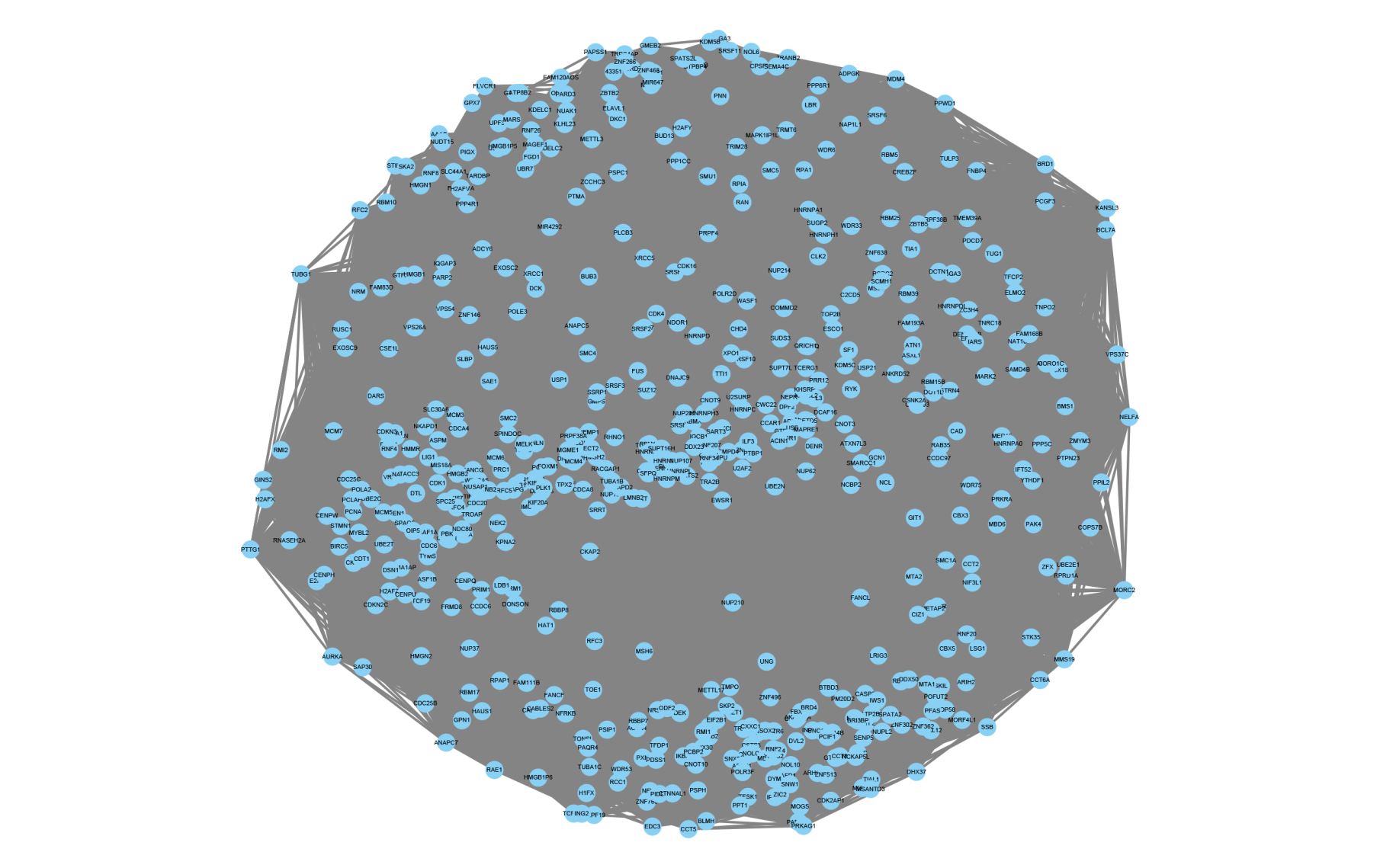


**Supplementary Fig. 5 Protein-protein interaction (PPI) network of genes in the turquoise module.**

PPI network was drawn using Cytoscape 3.6 software. Network nodes represent proteins and edges represent protein-protein associations.
